# Smoking and Unstable Plaque in Acute Coronary Syndrome: A Systematic Review of The Role of Matrix Metalloproteinases

**DOI:** 10.7150/ijms.79889

**Published:** 2023-02-13

**Authors:** Amilia Aminuddin, Siao Suan Cheong, Nur Aishah Che Roos, Azizah Ugusman

**Affiliations:** 1 Department of Physiology, Universiti Kebangsaan Malaysia Medical Centre, 56000 Cheras, Kuala Lumpur, Malaysia.; 2 Faculty of Medicine and Defense Health, National Defense University of Malaysia, 57000 Kem Sungai Besi, Malaysia.

**Keywords:** acute coronary syndrome, matrix metalloproteinase, smoking, atherosclerotic plaque rupture, cardiovascular disease

## Abstract

Smoking is a risk factor of acute coronary syndrome (ACS) that could increase matrix metalloproteinases (MMPs) levels, leading to unstable coronary artery plaque. The current review aimed to identify the relationship between smoking and MMPs in patients with ACS. Literature search was conducted from inception until March 2022 in three online databases. Risk of bias was assessed using the Newcastle-Ottawa Scale. A meta-analysis was performed, and the odds ratio (OR) together with its 95% confidence interval (CI) were determined. A total of 7,843 articles were identified, and only seven studies were included. Four studies investigated the MMP-3 and MMP-9 related genes and found that smokers with certain MMPs genotypes had high risk of ACS. Smoking also increased the MMPs level in patients with ACS compared with non-smokers. Additionally, a meta-analysis of two studies resulted in an increased odd of ACS in smokers with MMP-3 5A allele versus non-smokers with MMP-3 6A6A allele (OR: 15.94, 95% CI: 10.63-23.92; *I^2^*=55%). In conclusion, the current review highlights the role of MMPs in relation to smoking and ACS. The determination of these roles may help in identifying new ACS markers among smokers and the development of drug-targeted treatment.

## 1. Introduction

The prevalence of coronary artery disease (CAD) is increasing globally, particularly in developing countries. In Malaysia, ischemic heart disease remains as the principal cause of death among males and females in 2020 1. The underlying pathology of CAD is atherosclerosis, which involves the accumulation of fats, inflammatory cells, smooth muscles, and fibrous tissue at the intimal layer of the coronary artery wall. CAD usually presents with acute coronary syndrome (ACS), involving ST elevation myocardial infarction (STEMI), non-STEMI, and unstable angina (UA) 2. The most common cause of ACS is plaque rupture that leads to thrombus formation or emboli, and this condition reduces or blocks the perfusion of the heart 3. An easily ruptured plaque is also known as an unstable plaque 4. It is usually associated with a thin fibrous cap, increased necrotic core, lipid and macrophage content, high neovascularization and inflammatory mediators, and low smooth muscle cells and collagen content 3.

Plaque rupture may result from the activation of matrix metalloproteinases (MMPs) 5 that disrupt the mechanical integrity of plaque tissue 6. MMPs are a family of zinc-dependent endopeptidases that possess catalytic activity against extracellular matrix (ECM) components. This condition leads to the destabilization of the plaque fibrous cap, resulting in plaque rupture. MMP-1, MMP-2, MMP-9, MMP-12, and MMP-14 are involved in unstable plaques 5. Galis et al. (1994) found an increase in MMP-1 and MMP-9 in endothelial cells, vascular smooth muscle cells (VSMC), and other inflammatory cells, particularly at the susceptible region of human atherosclerotic lesions 7. The levels of MMP-1 and MMP-9 were significantly greater in human atheromatous plaques than in fibrous plaques 8. In the unstable carotid artery plaque, the levels of both MMP-2 and MMP-9 increased compared with that of the stable plaque 9. MMP-9 is an independent predictor of coronary plaque instability in CAD patients with increased lipoprotein(a) level 10, and it is related to poor cardiovascular disease (CVD) outcomes among patients with ACS 11.

MMPs are released by several cells such as macrophages, smooth muscle cells, and T lymphocytes. *In vitro* study showed that the formation of MMP-9 is triggered by several factors such as inflammatory mediators, including interleukin-β (IL-β) and tumour necrosis factor-α (TNF-α), oxidized low-density lipoprotein (LDL), lysophosphatidic acid (LPA), and phorbol myristate acetate (PMA), via several receptors such as Toll-like receptors (TLRs), IL-β, and TNF-α receptors of macrophage or lectin-like oxidized LDL receptor-1 (LOX-1) of endothelial cells 12, 13. Moreover, the stimulation of these receptors activates several transcription factors for MMP-9 release, such as activator protein 1 (AP-1) or nuclear factor kappa B (NF-κB). MMP-9 production increases with AP-1 in human coronary artery plaque 14. *In vitro* study by Zhou et al. (2018) demonstrated that the activation of NF-κB is required for the release of MMP-9 from monocytes 15. MMP-9 formation in macrophages was inhibited by interferon-γ (IFN-γ) 16, high-density lipoprotein (HDL) 12, AP-1 and NF-κB inhibitors 5, anti-inflammatory mediators such as IL-4 and IL-10 17, and tissue inhibitor of matrix metalloproteinases (TIMP). The sources of increased MMPs are the atherosclerotic plaque itself, damaged cardiac tissue, and circulating leukocytes or monocytes in the blood (peripheral blood mononuclear cells or PBMCs) 18.

Smoking is an important CAD risk factor that is frequently associated with unstable plaque 19. An epidemiology study found that among those with ACS, the prevalence of smoking was 37.1% and that of ex-smoker was 17%, which contributed to nearly 54% of the ACS cases 20. Although the underlying pathogenesis of smoking-induced atherosclerosis has not been completely recognized, modifications in the stability between production and degradation of extracellular matrix might be involved 21. Nicotine is an addictive component of cigarette smoke that induces the expression of MMP-2 and MMP-9, thus contributing to unstable plaque formation 22. Furthermore, Nordskog et al. (2003) reported that the exposure of human endothelial cells to cigarette smoke condensate upregulates the gene expression of MMP-1, MMP-8, and MMP-9 23.

Clinical research also supports the relationship between smoking and MMPs. Sivaraman et al. (2014) found increased MMP-2 and MMP-9 in patients with acute MI (AMI), and among the AMI patients, smokers had a higher level of MMP-9 compared with non-smokers 24. Huang et al. (2016) reported that MMP-1, MMP-9, and MMP-10 were significantly associated with current smoking habit in seniors without manifested CVD 25. Páramo et al. (2008) also found that both circulating MMP-1 and MMP-10 levels were significantly higher in smokers who were asymptomatic for CVD compared with non-smokers 21. This finding suggests that smoking might contribute to plaque instability via the action of MMPs 26. To gain more insight on this clinical knowledge, we aimed to investigate the association between smoking and MMPs in subjects with ACS and to understand its mechanism.

## 2. Methodology

This review was carried out in accordance with the Preferred Reporting Items for Systematic Reviews and Meta-Analyses guideline 27.

### 2.1 Search strategy

Literature search was conducted from inception until March 2022 among three online databases (PubMed, Ovid, and Scopus). Studies that were published until March 2022 were included. The keywords that were used for the search strategy were (coronary artery disease) OR (cardiovascular disease) OR (ischemic heart disease) OR (acute coronary syndrome) OR (coronary atherosclerosis) OR (myocardial infarction) AND (matrix metalloproteinase) AND (smoke). The reference list of the included studies was also screened for potential eligible studies.

### 2.2. Study Criteria

Articles that were obtained from the search were studied individually by two researchers (AA and SC). The inclusion criteria were as follows: (1) original papers available in English language, (2) studies that reported statistical comparison involving MMPs and smoking status, and (3) clinical studies involving adult human patients with CAD, who are both male and female regardless of ethnicity. Review articles, articles not in English, animal studies, and studies without statistical analysis involving MMPs and smoking status among CAD patients were excluded.

### 2.3. Article selection and data extraction

Article selection was organized in three stages by two independent researchers (AA and SC). Initially, papers were omitted primarily based on the title. Then, papers that were irrelevant to MMPs and smoking status were omitted by evaluating the abstracts. Lastly, papers that did not follow the inclusion criteria were omitted by evaluating the full paper completely. For data extraction, the name of the studies' first author, study characteristic, age and sex of the participants, method of MMP measurement, correlation/comparison between MMPs and smokers, and references were summarized in a table independently by two researchers (AA and SC). The comparison included the odds ratio (OR), and the value was presented in terms of median (interquartile range). For missing data, the author of the respective study was contacted by email to request for further information, if necessary.

### 2.4 Risk of bias assessment

Quality assessment for the risk of bias was conducted by two reviewers (AA and SC) by using the Newcastle-Ottawa Scale (NOS) for case-control and cohort study 28. The NOS tool for case-control study includes the assessment of the three following domains: (1) selection of study groups, (2) comparability between groups, and (3) ascertainment of exposure. The NOS tool for cohort study was used to assess the following domains: (1) selection of study groups, (2) comparability between groups, and (3) outcome assessment. The NOS tool consists of eight items within the three domains as described above. Each item was allocated a maximum of one star with maximum of two stars in the comparability item, where applicable. The maximum number of stars is nine, in which studies scoring 7-9 were considered of high quality, studies scoring 4 -6 were considered of fair quality, and studies scoring 1 -3 were considered of low quality. Any disagreement between the reviewers was settled by consensus or consultation with a third reviewer (CR).

### 2.5 Statistical analysis

A meta-analysis was performed using the Review Manager (RevMan) 5.4 software (The Cochrane Collaboration 2020). The OR together with its 95% CI was used in reporting the odds of CAD between smokers with MMP-3 5A5A+5A6A allele versus non-smokers with MMP-3 6A6A allele. The heterogeneity between studies was evaluated using (1) Chi-squared test with a p value of less than 0.10 to denote statistical significance and (2) the Higgin's *I^2^* statistic 29. An *I^2^* value of less than 25% was regarded as low heterogeneity, while an *I^2^* value of 75% or more was considered as high heterogeneity. Considering the small number of studies available for meta-analysis, a fixed-effect (FE) model was used. A p value less than 0.05 indicated statistical significance. Sensitivity analysis was conducted by inclusion or exclusion of study for the evaluation of result's robustness. No subgroup analysis was performed because of the limited number of studies. A funnel plot was not reported as less than 10 studies were included in the meta-analysis.

## 3. Results

**Figure 1:**
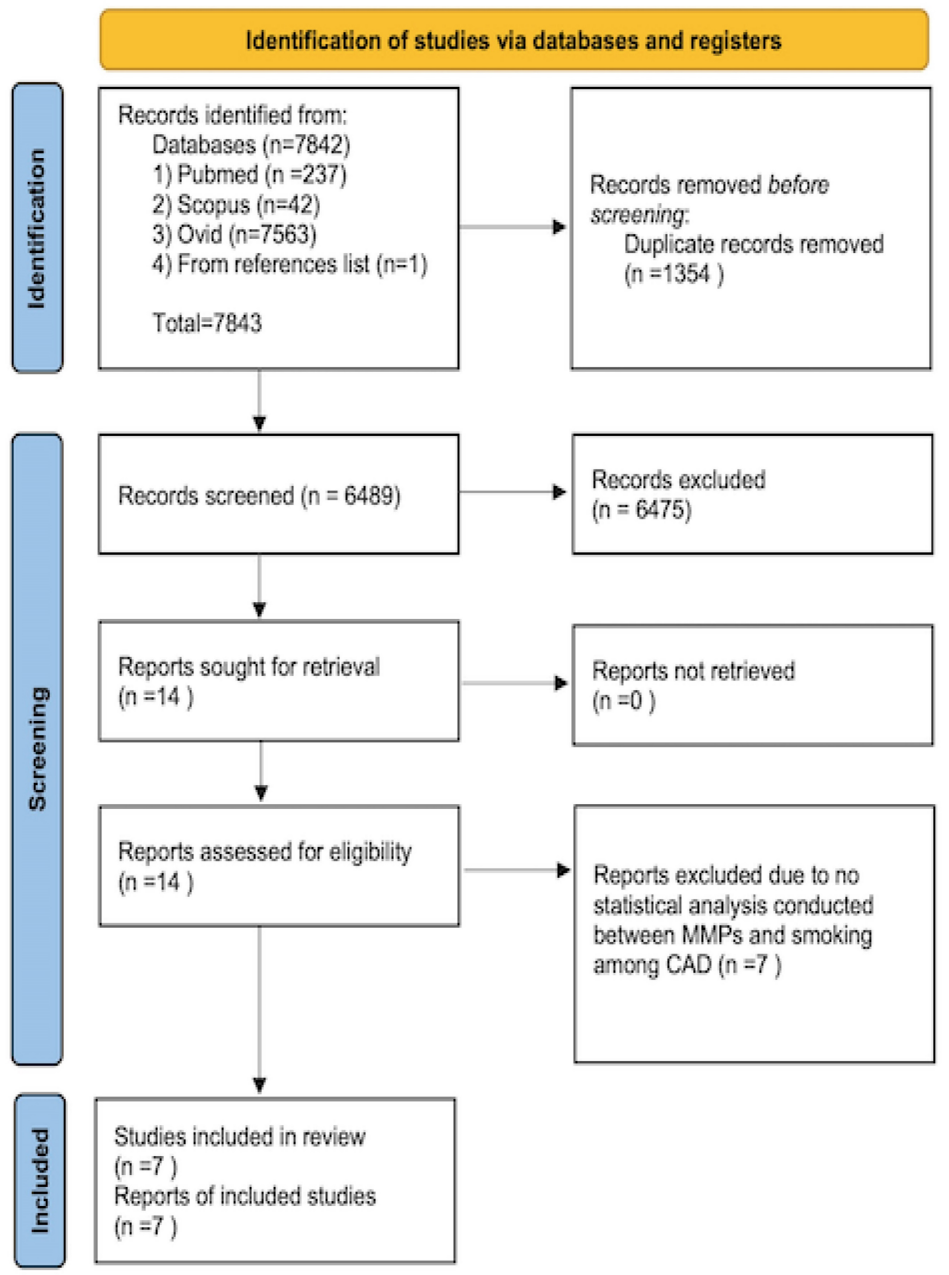
Flow chart of the article selection.

A total of 7,842 articles were obtained from three online databases, namely, Pubmed (237 articles), Scopus (42 articles), and Ovid (7,563 articles). One article was obtained by screening the included study reference list 30. The articles were published between 1961 and February 2022. A total of 1,354 articles were omitted because of replication. After assessing the titles and abstracts, 6,475 articles were omitted. The full articles for the 14 remaining were attained and evaluated completely. From these 14 articles, only seven articles were counted. The method of article collection is presented in Figure 1.

Table 1 summarizes the details of all seven studies. Six studies were case-control by design, and one study was a prospective cohort study. Based on the quality assessment, the NOS score range from 5 to 8 (fair to high quality). Most of the subjects were of middle to old age. Only one study involved young subjects with ACS 31. The MMPs measured were the salivary levels of MMP-8 and MMP-9 32 and the plasma levels of MMP-2 24, MMP-3 34, MMP-8 33 and MMP-9 24. Several studies have also investigated the MMP-related gene 30, 31, 33, 34. For example, Liu et al. (2003) 31, Liu et al. (2006) 34, and Humphries et al. (2002) 30 studied the MMP-3 related gene polymorphism (6A, 5A), while Wang et al. (2012) 35 investigated two single-nucleotide polymorphisms ([SNPs] -1562C>T and R279Q) of the MMP-9 gene.

For the interaction between MMPs and smoking status, Liu et al. (2006) observed that smokers who were carriers of the MMP-3 5A allele had a 20-fold higher risk of ACS compared with non-smoking patients and 6A/6A genotype carriers 34. Furthermore, the carriers of the MMP-3 5A allele, who are smokers, also had a 10-fold higher risk of young MI (OR: 9.98, 95% CI: 2.3-12.5) compared with 6A6A genotype carriers who are non-smokers 34. Humphries et al. (2002) found that smokers with MMP-3 5A5A genotype had 4.86-fold (95% CI: 2.04-11.56) higher risk for coronary heart disease (CHD) than non-smokers with a similar genotype 30. A significant genotype-smoking interaction was also observed (for 5A5A vs. other genotypes combined, p=0.04) 30. Wang et al. (2012) found that the interaction between the -1562 T allele of MMP-9 and smoking (OR: 4.42, 95% CI: 2.74-7.13; p<0.001) and the interaction between 279 Q allele and smoking (OR: 2.07, 95% CI: 1.04-4.13; p<0.021) were independent risk factors for MI 35. Lahdentausta et al. (2013) found that patients with AMI who are smokers had a higher MMP-8/TIMP ratio than non-smokers with AMI 33. Increased MMP/TIMP ratio produced unstable plaque 33. Sivaraman et al. (2014) found that the MMP-9 level increased in smokers with AMI compared with non-smokers 24. By contrast, Rathnayake et al. (2015) showed that salivary MMP-8 and MMP-9 levels did not significantly differ between smokers and non-smokers with AMI 32.

**Figure 2:**
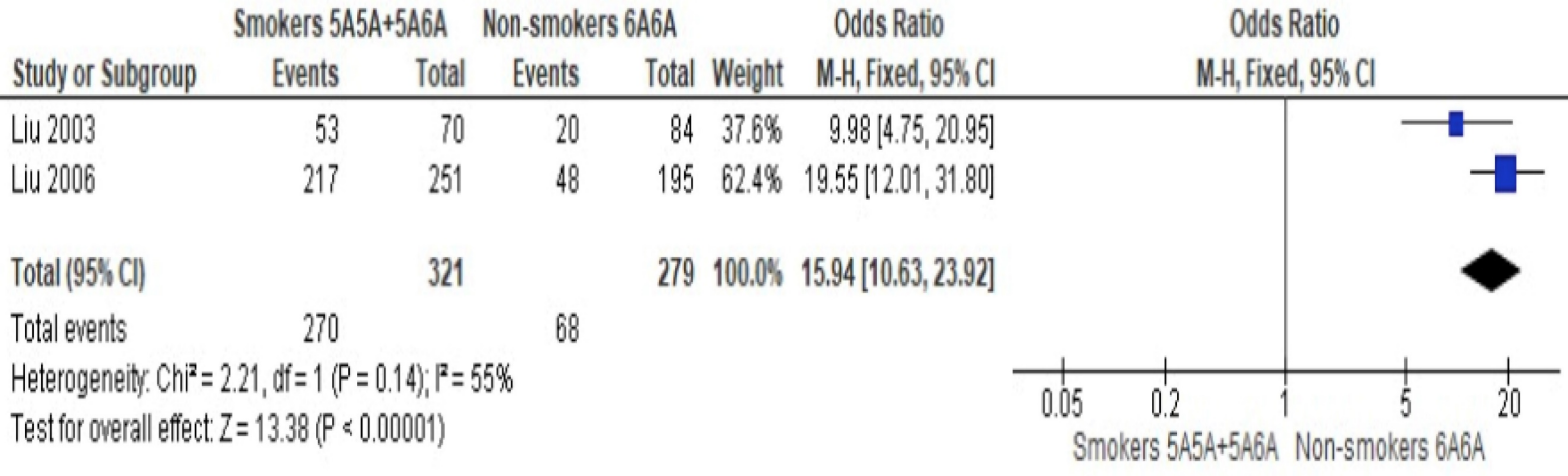
Meta-analysis of the odds of acute coronary syndrome between smokers with MMP-3 5A allele versus non-smokers with MMP-3 6A6A allele.

Altogether, only two studies have provided suitable data to be pooled together. In a meta-analysis of two studies, Liu et al. (2003) 31 and Liu et al. (2006) 34 obtained an OR of 15.94 (95% CI: 10.63-23.92; p<0.01; *I^2^*=55%) and found that smokers with MMP-3 5A allele had significantly higher odds of ACS than non-smokers with MMP-3 6A6A allele (Figure 2). The obtained heterogeneity was substantial. A sensitivity analysis was conducted by including a study of Humphries et al. (2002) 30, which is a cohort study with an OR of 6.16 (95% CI: 4.49-8.46; p < 0.01; *I^2^* = 97%). Although the resulting OR and 95% are precise, the significantly high heterogeneity observed indicated that the pooling of studies together is not appropriate. High heterogeneity observed possiby because of the differences in population studied (MI vs. CHD events).

## 4. Discussion

This review aimed to identify the association between smoking and MMPs among patients with ACS. Based on the limited data, smoking has a positive association with MMP genes and levels. This finding may explain the damaging effect of smoking on blood vessel through the formation of unstable atherosclerotic plaque and plaque rupture, causing ACS. By contrast, only one study did not support such finding 32. This phenomenon can be attributed to the nature of the study that examine the saliva sample compared with other studies that measured MMPs in the blood.

Atherosclerosis is mainly involved in a native immune reaction and is characterized by chronic inflammatory process in the vessel wall. Cigarette smoke is fundamental to the broad effects of smoking on vascular pathology by inducing vascular inflammation and oxidative stress 36. Cigarette smoke exposure may lead to excessive MMP activity and inflammation. Elevated MMP activity is responsible for plaque activation and disruption 24, 37.

From *in vitro* and *in vivo* studies, nicotine, which is the main compound involved in cigarette, stimulates the expression of several MMPs. Ren et al. (2018) observed that nicotine administration to atherosclerotic apolipoprotein E-deficient (ApoE^-/-^) mice increased the area of the atherosclerotic lesions with increased collagen, lipid, and macrophage content in the lesion 22. Nicotine also increased the levels of circulating inflammatory cytokines (IL-6 and TNF-α), MMP-2, and MMP-9 levels in the atherosclerotic plaque. Both changes were blocked by giving α1‐AAV, which inhibited the expression of α1-nicotinic acetylcholine receptors (α1-nAChRs). These findings suggest that the effect of nicotine on atherogenesis is through α1-nAChRs via the MMP-2/MMP-9 pathway 22. In another study, Florence et al. (2017) reported that leukocytes derived from ApoE^-/-^ mice exposed to secondhand smoke released a high amount of MMP-9 38. MMP-9 activates protease activated receptor-1 (PAR-1), which exerts pro-inflammatory, pro-apoptosis, and pro-atherogenic effects on endothelial cells. Inhibiting MMP-9 expression and production successfully reduced the atherosclerotic changes and improved the overall vascular health of the mice. These findings affirm the effect of MMP-9 in atherogenesis 38.

The production of MMPs secondary to nicotine or cigarette smoke exposure involves several pathways. Liu et al. (2014) found that nicotine increased MMP-2 and MMP-9 levels via the extracellular signal-regulated kinase1/2 (ERK1/2) signalling pathway through the activation of transcription factor AP-1 in mouse aortic smooth muscle cells 39. ERK1/2 is part of the mammalian mitogen-activated protein kinase (MAPK) family and is involved in numerous cellular actions, such as cell multiplication, differentiation, and existence 39. The transcription factor AP-1 family includes multiple Jun (c-Jun, JunB, and JunD) and Fos (c-Fos, FosB, Fral, and Fra2) members. The AP-1 family is involved in several physiological and pathological cellular activities, such as proliferation, inflammatory process, differentiation, development, migration, and cell death 40.

Palozza et al. (2012) showed that cigarette smoke-mediated MMP-9 expression was reduced by preventing the prenylation of Ras, involving MEK1/2-ERK1/2 and NF-κB signalling 41. The prenylation of Ras protein in macrophage or fibroblast is inhibited by lycopene by reducing the expression of HMG-CoA reductase and inhibiting mevalonate production. Lycopene also inactivates Ras protein by impairing the localization of Ras, causing a cytoplasmic increase in Ras. Ras stimulation is the initial stage of MAPK cascade activation for MMP-9 induction, while MMP-9 induction involves ERK1/2 phosphorylation. The binding action of NF-κB is also modulated by lycopene by regulating the pathways that converge at NF-κB binding sites 41. The secretion of MMPs caused by nicotine exposure also enhances oxidative stress. Thompson et al. (2011) showed that the activation of MMP-2 by prenatal nicotine exposure triggered oxidative stress, thus contributing to the degradation of matrix components and accumulation of interstitial collagen in fetal guinea pig heart 42. This condition was reduced by giving N-acetylcysteine (NAC), which is an antioxidant. Khoi et al. (2013) showed that nicotine stimulated MMP-9 gene expression by inducing intracellular reactive oxygen species (ROS) formation and activation of NF-κB and AP-1 transcription in human ECV304 endothelial cells 43. Interestingly, (-)-epigallocatechin-3-gallate (EGCG), which is the main compound green tea, and it suppresses MMP-9 expression by inhibiting ROS formation and the activation of NF-κB and AP-1. EGCG inhibits AP-1 transcriptional activity by suppressing c-fos and c-jun activation, which is the downstream transcriptional target of JNK and ERK1/2 pathways. AP-1 activity could also be inhibited by EGCG through the direct inhibition of MAPK activities, because EGCG inhibits the phosphorylation of receptor tyrosine kinase 43.

Apart from nicotine, acrolein is an aldehyde constituent that has been discovered in cigarette smoke at increased concentration 44. Treatment of human aortic endothelial cells in culture with acrolein leads to the upregulation of MMP-1 expression and downregulation of TIMP-3 through the inhibition of the mammalian target of the mTOR/p70S6K pathway. Both MMP-1 and TIMP-3 are the main modulators of angiogenesis and impair neovascularrisation in diseased vessel wall by affecting the integrity of the surrounding ECM 44. In another study, the exposure of PMA-differentiated THP-1 cells to acrolein induced MMP-9 production 45. This process was achieved by increasing the intracellular calcium level, which activated xanthine oxidase and ROS production in the macrophage cell line. The upregulation of MMP-9 in acrolein-treated macrophages was attenuated by xanthine oxidase inhibitor that inhibited ROS generation. Moreover, acrolein stimulated MMP activity in advanced atherosclerotic lesions of ApoE^-/-^ mice, which could destabilize the lesion 45.

Smoking is an important CAD risk factor, it interacts with several genes that may enhance the risk of ACS. Several other genes that are not related to MMPs have been implicated, such as lipoprotein lipase-9N variant 46, the E4 variant of the apolipoprotein E gene 47, polymorphism of glutathione-S-transferase with M1 isoform 48, and G33A polymorphism in thrombomodulin gene 49. Based on the current review, smoking has a synergistic effect with MMP gene polymorphisms, such as MMP-3 5A allele and MMP-9 1562C>T locus with CT or TT genotype. Studies on the interaction between MMPs related genes, smoking, and ACS are limited, thus requiring further studies. Identifying such interaction may help in preventing future ACS, especially among smokers.

Several studies have been conducted to determine the effects of MMP inhibition on the atherosclerotic plaque. Most of the studies involve preclinical *in vitro* and *in vivo* studies, and no clinical trials have been carried out in the past. The preclinical studies involve several methods, such as the use of direct MMP inhibitors 50, 51, MMP knock out mice 52, 53, 54, or overexpression of TIMP 55, 56. For the use of MMP inhibitors, Johnson et al. (2011) demonstrated that treatment of atherosclerotic apolipoprotein E-knockout mice (ApoE^-/-^) with RXP470.1 (MMP-12 inhibitor) successfully reduced the cross-sectional area of the atherosclerotic plaque 50. The ratio of smooth muscle cell to macrophage increased, macrophage apoptosis decreased, cap thickness increased, and necrotic cores and calcification decreased in the plaque. Additional *in vitro* and *in vivo* studies showed the attenuation of monocyte/macrophage invasion and reduction in macrophage apoptosis. By contrast, the treatment of ApoE^-/-^ mice with a broad-spectrum MMP inhibitor, RS-130830, increased the lipid content and decreased the collagen content of their brachiocephalic atherosclerotic plaque, which favours plaque instability 51.

In another study that involves double MMP and ApoE knockout mice, Laxton et al. (2009) found that MMP-8^-/-^/ApoE^-/-^ mice had less aortic atherosclerosis than the control mice 52. The plaque lesion also had a significantly less macrophage, a lower trend of smooth muscle cells, and a higher collagen content. Similarly, Kuzuya et al. (2006) found that MMP-2^-/-^/ApoE^-/-^ mice had less atherosclerotic plaque in their aortic sinus and arch with decreased smooth muscle cell-positive area than MMP-2^+/+^/ApoE^-/-^mice 53. Interestingly, Johnson et al. (2005) found divergent effects of MMP-3, MMP-9, MMP-7, and MMP-12 in ApoE/MMP double knockout mice's atherosclerotic plaque 54. In ApoE^-/-^/MMP-3^-/-^ and ApoE^-/-^/MMP-9^-/-^ mice, the brachiocephalic artery plaque was larger than that of the control with a higher number of buried fibrous layers. The ApoE^-/-^/MMP-3^-/-^ and ApoE^-/-^/MMP-9^-/-^ mice also exhibited cellular compositional changes, indicating an unstable plaque phenotype. By contrast, the plaque lesion in ApoE^-/-^/MMP-12^-/-^ mice was reduced, with increased smooth muscle cell and reduced macrophage content in the plaque, indicating a stable plaque phenotype. Atherosclerotic lesion in ApoE^-/-^/MMP-7^-/-^ mice showed no difference from that of the control mice 54.

Among the studies that focused on MMP suppression by TIMP overexpression, Johnson et al. (2006b) found that TIMP-2 overexpression in ApoE^-/-^ mice reduced the smooth muscle cell and macrophage content in the atherosclerotic plaque and the presence of buried fibrous caps 55. An *in vitro* study has revealed that treatment of macrophages and macrophage-derived foam cells with exogenous TIMP-2 significantly inhibited migration and apoptosis of macrophages and foam cells 55. In addition, Rouis et al. (1999) found that mice infused with rAd.RSV.TIMP-1, which increased TIMP-1 plasma level, had a marked reduction in atherosclerotic lesion compared with rAd.RSV.βGal-infused mice 56. The lesion also had reduced macrophage deposition and MMP-2, MMP-3, and MMP-13 levels.

Apart from causing plaque instability that leads to ACS, MMPs are also involved in the formation of plaque erosion. Plaque erosion has a low level of plaque vulnerability because of the intact fibrous cap, but it is becoming an increasingly common characteristic of a culprit lesion in ACS and is responsible for one-third of ACS 57. Plaque erosion occurs without fibrous cap disruption, where blood comes into contact with an intimal surface that lacks endothelial cells 58. Changes in endothelial shear stress gradients initiates plaque erosion by activating the Toll-like receptor 2 (TLR2) in endothelial cells. TLR2 activation causes a type of smouldering inflammatory response that compromises the integrity of the basement membrane and induces cytoskeletal reorganization in the endothelial cells by reducing the vascular endothelial cadherin levels. Both MMP-2 and MMP-9 contribute to endothelial-to-mesenchymal transition (EndMT), resulting in the desquamation of the endothelial cells from the basement membrane and subsequent plaque erosion. Subsequently, neutrophil extracellular traps facilitate platelet-rich thrombus formation at an area of high shear stress that propagates distally with the blood flow and settles until it reaches an area of high oscillatory shear index 57. An eroded plaque is likely to develop occlusive or non-occlusive thrombus that could easily be embolized because of its relatively preserved vascular structure 59. Smoking is also an important risk factor for plaque erosion, especially in females 60, 61.

Based on the present review, no clinical study has been conducted to determine the effects of smoking abstinence on MMP levels among patients with ACS. Nevertheless, the effect has been investigated in other conditions such as wound healing 62, healthy subjects 63, 64, 65, and chronic obstructive pulmonary disease 66. Their findings varied, in which some studies found that the levels of MMP decreased 62, 63, 64, while other studies found that the levels were increased 66 or unchanged 62. The difference can be attributed to the different types of subjects, the types of MMP studied, the associated illness, and the duration of abstinence.

**Figure 3:**
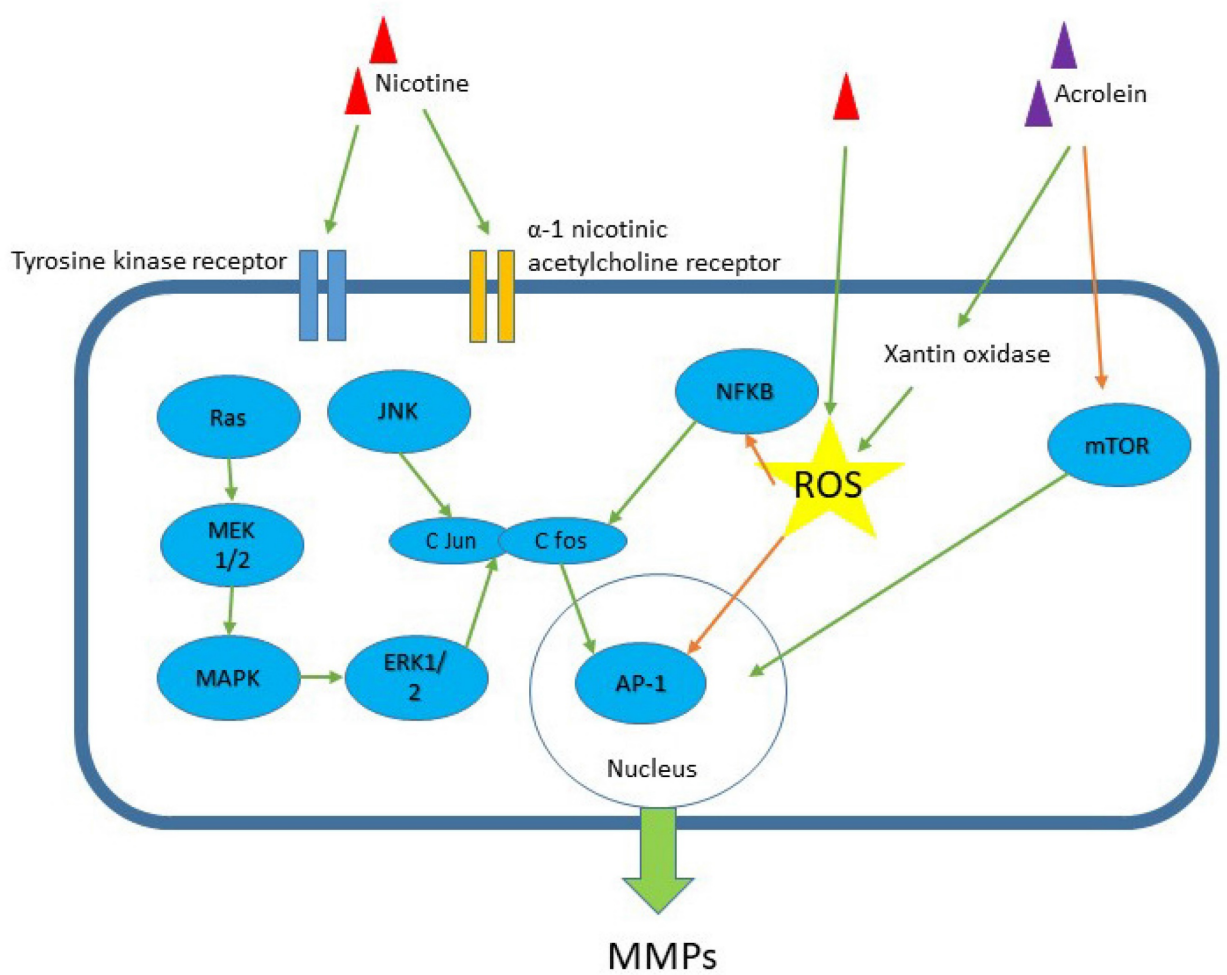
Signalling pathway for nicotine-induced MMP secretion in vascular and inflammatory cells models. Among the components of cigarette smoke are nicotine and acrolein. The receptors recognized include tyrosine kinase and α-1 nicotinic acetylcholine receptor. Several pathways have been implicated such as MAPK, JNK, ERK1/2 and mTOR pathways, and these pathways activate transcription protein AP-1 and NF-kB. Green arrow - stimulates; orange arrow - inhibits. ROS = reactive oxygen species.

Although the current review supports the close association between MMPs and smoking in ACS, the use of MMPs as biomarkers in predicting ACS could be masked by smoking history. Lahdentausta et al. (2013) found that smoking reduced the association of MMP-8 with ACS 33. They found that smoking reduces the diagnostic capacity of MMP-8 and MMP-8/TIMP-1 ratio in ACS. Future studies should establish this issue. Nevertheless, cigarette smoking induces vascular inflammation, endothelial dysfunction, and vascular remodelling for coronary events, which are facilitated by the upregulation of MMP activities 24. Figure 3 summarizes the relevant pathways related to the components of cigarettes smokes and MMPs. The presumably pathway mechanisms of how smoking induces MMPs secretion that cause matrix remodelling in ACS is a topic of interest in the near future. Exploring these pathways may help in identifying new ACS markers and the development of drug-targeted treatment.

## 5. Conclusions

In conclusion, based on the limited data, smoking has a close relationship with MMPs that can lead to plaque instability and ACS. Identifying the pathways involved, the type of MMPs with a marked association with ACS, the genetic contribution, the change in MMP levels following smoking abstinence, and clinical trials on the effects of MMP inhibition are among the areas that can be further explored. The findings may improve the understanding about the role of MMPs among smokers with increased risk of atherosclerosis, which could help in ACS prevention and the development of drug-targeted therapy.

## 6. Study Limitation

In this review, several limitations have been identified. Firstly, MMPs are a heterogeneous group of enzymes and the different studies discussed in this review have targeted different MMPs. Some studies looked at MMP-3, some studies focused on MMP-8 and-9, while others evaluated the MMP/TIMP ratio. Secondly, the studies are also heterogeneous with some studies measuring MMP levels in the blood, some studies measuring MMP levels in the saliva, while others looked at the gene polymorphisms rather than the blood levels of MMPs. Thirdly, most studies had a modest sample size between 125 to 650 CHD subjects.

## Figures and Tables

**Table 1:** Summary of the findings from the selected studies.

References	Study design, subject characteristics, and male %	Mean age (years)	Methods and MMPs measured	Definition of smoker	Correlation between MMPs and smoking status or comparison	NOS score
Sivaraman et al. (2014) 24	Case control study. 300 AMI patients and 100 age- and sex-matched control 222 AMI patients with the habit of smoking 78 non-smokers with AMI 62 normal controls with the habit of smoking 38 normal controls without smoking	NS	NS	NS	MMP-9 was significantly (p<0.01) elevated in AMI patients who smoked when compared with AMI patients who are non-smokers.	7
Humphries et al. (2002) 30	Prospective study 2743 middle-aged men, who are free of CHD at baseline, were followed up for 24,000 person-years. 125 patients experienced CHD event, while 348 were control. Only Caucasian individuals were included.	Control= 56.8 (3.5) CHD event= 56.9 (3.6)	Genotyping of MMP-3 was carried out by using a universal heteroduplex	Never smokers, ex-smokers if they reported a smoking history but had quit for at least 6 months at recruitment, and current smokers.	Smokers with 5A5A genotype had 4.86-fold (CI 2.04-11.56) higher risk than in non-smokers with this genotype, with significant evidence for a genotype-smoking interaction (for 5A5A vs. other genotypes combined, p=0.04).	8
Liu et al. (2003) 31	Case control study 150 patients with MI age <45 years old Control: 83.5% male AMI: 84% male	Control: 44.51±7.85 AMI: 43.95 ±5.33	Genotype stromelysin-1 (MMP-3) promoter was determined using polymerase chain reaction and direct sequencing	Current or ex-smokers and non-smokers.	Smokers with the 6A6A genotype was associated with a 3-fold higher risk for young MI (OR 3.09, 95% CI 1.2 to 7.6). Furthermore, smokers with the stromelysin-1 5A allele had a significantly 10-fold higher risk of young MI (OR 9.98, 95% CI 2.3 to 12.5) compared with non-smokers and 6A6A genotype carriers.	5
Rathnayake et al. (2015) 32	Case control study. 200 patients with a first MI. 200 controls matched for age, gender, residential area, and without previous MI.	MI: 61±8 years Non-MI: 61±8 years	The biomarkers MMP-8, MMP-9, and TIMP-1 were analyzed by time-resolved immunofluorescence assay (IFMA), Western blot, and ELISA.	NS	No significant differences were observed in MMP-8, MMP-9 level and MMP-8/TIMP ratio between smokers and non-smokers with MI.	6
Lahdentausta et al. (2013) 33	Case-control population of 605 who were age under 80 years and without signs of cognitive intellectual disability. 291 ACS: Acute MI (*n*=197) or UAP (*n*=94). 314 healthy control individuals. ACS and smoking (*n*=55). Control and smoking (*n*=66).	Cases: Smoking ACS: 56.8±9.38 Non-smoking ACS: 64.2±8.33 Controls: Smoking controls: 61.9±8.25 Non-smoking controls: 63.4±9.57	Serum MMP-8 concentrations were determined via time-resolved immunofluorometric assay (IFMA). TIMP-1 concentrations were determined using ELISA.	NS	The smoking AMI patients had higher MMP-8/TIMP-1 ratio [0.43(0.97)] compared with non-smokers having AMI [0.34 (0.42)].	6
Liu et al. (2006) 34	Case control study. 650 consecutive Taiwanese patients diagnosed with acute coronary syndrome Control (*n*=350), 78% male. UAP (*n*=200), 78% male. NSTEMI (*n*=200), 75% male. STEMI (*n*=350), 79% male.	Control: 57.5±7.8 UA: 55.5±7.4 NSTEMI: 56.8±9.5 STEMI: 53.7±6.2	Direct sequencing on the 5 adenines (5A)/6 adenines (6A; 1,171 bp) polymorphism in the MMP-3 gene promoter region	NS	Smokers carrying the 6A/6A genotype were associated with a 9.3-fold higher risk for ACS. Furthermore, smokers who were carriers of the MMP-3 5A allele had a 20-fold higher risk of acute coronary syndrome compared with non-smokers and 6A/6A genotype carriers.	7
Wang et al. (2012) 35	Case control study. A total of 835 Uighur participants who were genetically unrelated 384 coronary angiography-proven MI patients (acute MI or NSTEMI) 451 sex-matched and ethically matched control participants	MI group: 55.6 ± 10.9 years Control group: 54.1± 10.3 years	Genotypes of two selected SNPs (-1562C>T and R279Q) of the MMP-9 were determined via polymerase chain reaction and restriction fragment length polymorphism (PCR-RFLP)	Smokers: consuming more than 5 cigarettes per day Non-smokers: never smoked or had stopped smoking at least 1 year before sample collection	At -1562C>T locus, compared with the CC genotype and nonsmokers, smokers carrying mutant T allele (CT or TT genotype) showed significantly higher risk (OR = 3.64, 95% CI: 2.04-6.49) of MI. Among the individuals who carry CC genotype, smokers had 1.8-fold risk of MI (OR=1.81, 95% CI: 1.32-2.49. In comparison with the non-smokers with -1562 CC genotype, smokers having the -1562 CT or TT genotype were associated with a 1.31-fold risk of MI. Multiple logistic regression analysis showed that the interaction between smoking and -1562 T allele significantly increased MI risk by 4.42-fold. Smokers with 279Q allele (RQ or QQ genotype) was a significant risk factor for MI. The interaction between R279Q polymorphism and smoking was higher than the independent effect of smoking.	7

ACS= acute coronary syndrome; MI= myocardial infarction; MMP= matrix metalloproteinase; MPO= myeloperoxidase; NOS= Newcastle-Ottawa Quality Score; NS=not stated; NSTEMI= non-ST elevation MI; STEMI= ST elevation MI; UAP= unstable angina pectoris; TIMP= Tissue inhibitor of matrix metalloproteinases.
